# Cost-effectiveness of screening mammography in a low income country: a Markov simulation analysis

**DOI:** 10.1186/s12880-021-00696-z

**Published:** 2021-11-02

**Authors:** Segni Kejela

**Affiliations:** College of Health Sciences, Addis Ababa University, Addis Ababa, Ethiopia

**Keywords:** Markov multistate analysis, Low income countries, Screening mammography

## Abstract

**Background:**

Breast cancer is the most common cancer diagnosed in women. Screening mammography is the only imaging screening study for breast cancer with a proven. mortality benefit. This study aims to analyze the cost-effectiveness of screening mammography in Ethiopia.

**Methods:**

Multistate Markov model was used for computer simulation to estimate cost and health benefits of screening mammography interventions for age-group of 40–49 years and 50–59 years. The cost-effectiveness analysis was made for 4 policies based on where the screening mammography procedures were conducted: government institution only, the private institution only, 50% ratio for each, and 10% private institution policy. Outputs were expressed in total cost, life-years gained (LYG) incremental cost-effectiveness ratio (ICER), and incremental net monetary benefit (INMB).

**Results:**

All 4 policies of annual screening mammography failed to achieve acceptable ICER and lead to a net loss in INMB. The lowest ICER value was for government institution-only policy with 3510.3 USD/LYG and 3224.9 USD/LYG both above the cost-effectiveness threshold of 2808.5 USD. The cost per single death averted for each group was 110,206.7 USD and 77,088.2 USD for age-group 40–49 years and 50–59 years respectively.

**Conclusion:**

Screening mammography could not be shown to be cost-effective in Ethiopia with the current low cost-effectiveness threshold. Alternative screening approach like annual clinical breast examination may need to be investigated.

## Introduction

Breast cancer is the most common cancer among women worldwide. Globally breast cancer contributes to 24.2% of all cancer diagnoses and 15% of cancer mortality [1, 2]. Majority of this burden occurs in low and middle-income countries with more than 60% global annual mortality being recorded in this segment of the world with the reported highest mortality rate from Africa [3, 4]. This is likely from poor resources and manpower compounded by the delay in presentation and confounding traditional factors [5, 6].

Screening mammography is the only imaging screening study for breast cancer with a significant mortality benefit that the National Comprehensive Cancer Network (NCCN) has put forward as a category 1 protocol for patients with average risk with age 40 years or above [7, 8]. Similar clinical practice guidelines have been published from eastern countries with similar annual mammography screening recommendations though few maintained biennial frequency [9, 10].

African nations' recommendations are generally lacking even in more developed countries like South Africa [11]. Considering lower median age at diagnosis in the Sub-Saharan African population of breast cancer patients with possibly higher resultant aggressive nature of breast cancer in young, it seems imperative to look into the possibility of screening mammography from 40 years onwards [12, 13].

In Ethiopia as in all African nations, no national breast cancer screening program is present. Nationally mammography services are available, albeit not widespread, both in governmental and private health institutions. No national data is to date available on the proportion of breast cancer patients diagnosed with screening mammography and the number of patients currently enrolled in the screening program. Additionally, the cost-effectiveness and feasibility of a national breast cancer screening program have not been studied in Ethiopia or African nations. This study aims to analyze the cost-effectiveness of the annual national screening mammography program for all Ethiopian women age 40 years and above.

## Methods

### Method description

**Fig. 1:**
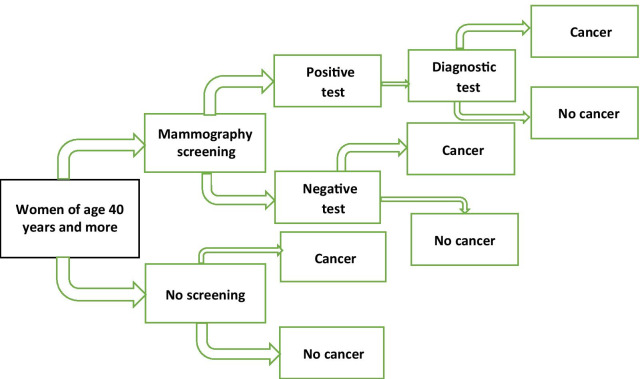
Decision tree model

Screening mammography is used to detect breast cancer at an earlier stage than the non-screened population group. Screening mammography has age-specific sensitivity and specificity, so there are a group of patients diagnosed with symptoms as in a non-screened population while on screening protocol and likewise, some patients will undergo diagnostic tests because of false-positive screening mammography results. All diagnosed breast cancer patients undergo treatment based on their respective stage, grade, the genetic and immunochemical status of the tumor. All these steps result in predictable cancer progression stages on which simulation models can be conducted (Fig. 1).

**Fig. 2:**
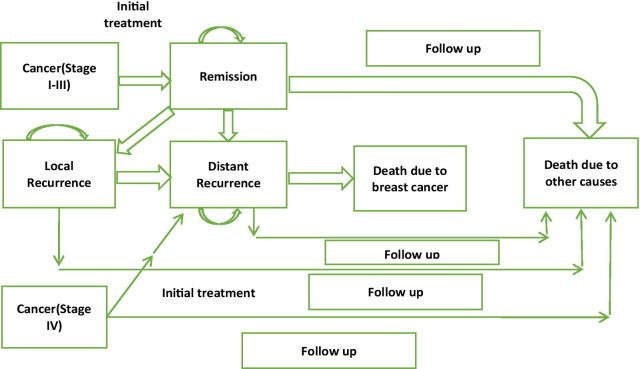
Health and transition states

Age categories of 40–49 years and 50–49 years were chosen and the assumption of 100% participation in the screening program was made for the simulation. The age group 30–39 years were excluded because of the absence of validated screening protocol in this age group and the age group 60–69 years were excluded because of the lower life expectancy of the Ethiopian population and resultant lower contribution to the overall population number [13]. The cost and death averted by screening were compared to the population with no screening. All women suspected of breast cancer from the screening group had incurred additional costs of the diagnostic test. Diagnostic test of FNAC was chosen for all suspected and for all non-screened patients as core needle biopsy is not widely available in Ethiopia. All screening or non-screening detected breast cancer cases were assumed to have undergone proper treatment for the respective stages. Women in each of diagnosed breast cancer were enrolled into the Markov chain extended from the decision tree (Fig. 2).

The Multi-state Markov model was used for analysis to incorporate multiple states of the output of health states. 5 states were chosen, Remission, local recurrence, distant recurrence, death from breast cancer, and death from other causes. Local and distant recurrences were assumed to remain in the same stage or progress. Outputs for the models were, cost, life years, cost per death averted, and the number of women screened for death averted (Fig. 2).

### Model inputs

#### Epidemiologic inputs

Table 1 depicts all essential inputs used in this model. Age distribution of breast cancer was extrapolated from the proportion of breast cancer patients by age group from Kantelhardt et al. and extrapolated to the whole population from both the national population proportions and WHO cancer data for breast cancer for Ethiopia, and the final output of breast cancer incidence was calculated and presented [13, 14]. The transition between health states for breast cancer is not available for Ethiopia, and Gocgun et al. data from their Markov model was utilized [15]. All patients, screened or non-screened diagnosed with breast cancer were assumed to have the same transition probabilities.

**Table 1:** Essential inputs for the model

Name	Live value		Distribution	Reference
Age distribution of Breast cancer			Beta	Kantelhardt, et al. [14]
30–39		0.000853		
40–49		0.000878		
50–59		0.00079		
60–69		0.000656		
Stage progression transition probabilities				
Stage I	Age 40–49	Age 50–59	Beta	Gocgun et al. [15]
Remission to local recurrence	0.01	0.009		
Remission to distal recurrence	0.000016	0.000025		
Local recurrence to distal recurrence	0.062	0.052		
Local recurrence to cancer death	0.013			
Distal recurrence to cancer death	0.555	0.137		
Stage II & III			Beta	
Remission to local recurrence	0.018	0.016		
Remission to distal recurrence	0.024	0.105		
Local recurrence to distal recurrence	0.165	0.13		
Distal recurrence to cancer death	0.386	0.423		
Stage IV			Beta	
Distal recurrence to cancer death	0.386	0.423		
Mammography sensitivity and specificity (40–49, 50–59 years)			Beta	Keen et al. [16]
Sensitivity		0.821, 0.921		
Specificity		0.859, 0.859		
Breast cancer stage distribution, screened			MBT	Wong et al. [17]
Stage I		0.521		
Stage II		0.382		
Stage III		0.057		
Stage IV		0.041		
Breast cancer stage distribution, Non-screened			MBT	Tesfaw et al. [18]
Stage I		0.1		
Stage II		0.189		
Stage III		0.569		
Stage IV		0.143		
FNAC(USD)		22	Invariant	Personal communication
Cost of mammography(USD)			Invariant	Personal communication
Government Institutions		4.5		
Private institutions		42		
Doctor visit cost		6		
Treatment cost(USD)			Gamma	
Stage I		160		Hoang Lan, et al. [19]
Stage II		458.48		
Stage III		850.45		
Stage IV		668.7		
Expected life, 40–44		36.10367697	Gamma	World Health Organization [19]
Expected life, 45–49		31.72113989	Gamma	World Health Organization [19]

*FNAC* fine-needle aspiration cytology, *MBT* multivariate beta distribution, *USD* United States Dollars

There is no publication available on breast cancer screening in Ethiopia. For this reason, screening mammography sensitivity and specificity for age were extracted from Keen et al. for both age groups [16]. In certain occasions of uncertainties in the images, a fuzzy preprocessing would be necessary [20]. Similarly, stages at diagnosis of breast cancer for a screening-detected group of patients were not nationally available and was extrapolated from the Chinese screening program [17]. But, stage at diagnosis for non-screened patients for Ethiopia was available from Tesfaw et al. [18].

The price for mammography and FNAC is set by institutions in both private and government institutions. For this reason, both major government and private institutions were communicated and the mean reported prices for each category of service were used and translated to United States Dollars (USD).

Treatment cost was only available in the form of cost per patient treated for breast cancer in an Ethiopian oncology center with no Stage-specific report. With the cost of 893 USD in direct medical costs, and accounting for a higher stage III proportion of patients in the Ethiopian non-screened group, the medical costs of Stage III breast cancer from the Vietnam study were well nearly equal to the average cost of breast cancer treatment in Ethiopia. So, the price report of Hoang Lan and colleagues was utilized for estimating the treatment price [19, 21].

WHO life tables used for expected life years loss and gain analysis for each age-group category specific to Ethiopia [22]. Discount rates of 5% for cost and 3% for life years were used for this model.

### Outputs

The two price points were analyzed separately first for total cost and final incremental cost-effectiveness ratio (ICER). Then assumption was made in which the two institutions shared 50% of screening mammography and similarly total cost and ICER was again calculated. A similar model was generated for a government-dominated screening with 90% and the rest 10% screened at a private institution. Total life-years gained from screened, the number of cancer screened to avert one death, and cost per death averted were calculated for each price category. Specific willingness to pay amount has not been set for screening mammography in Ethiopia. The mammography screening is considered cost-effective if the ICER was less than 3 times the GDP per capita according to the WHO recommendation which was determined to be 2808.9 USD from the 2020 world bank report [23, 24]. Incremental net monetary benefit (INMB) was calculated for ICER outputs that are found to be less than a willingness to pay threshold (3*GDP per capita).

### Sensitivity analysis

One-way sensitivity analysis was done using all likely parameters, and values that affected the output were presented on a tornado plot for both age groups analyzed in this model. Second-order Monte Carlo simulation was conducted using 10,000 iterations. All values except discount rates were set to have low and high values of 20% less or more than the base case values respectively because of conclusive data for specific value ranges. Discount rates lowest range of 0% and high value of 6% were utilized for sensitivity analysis.

### Ethical considerations

This is a simulation analysis involving no human or animal subjects. Only publicly accessible information was utilized for the simulation creation. With no human subject involved the study is not subject to any ethical consideration from Helsinki declarations and National Health Research Guidelines.

## Results

Table 2 summarized the three screening policies separately with the respective total cost, ICER, life-years gained, cost per death averted and number screened per death averted. All results on the table are presented per 100,000 women over 10 year period. The standard errors of total costs were 751.41 and 762.720 for age-group 40–49 years and 50–59 years respectively. 78.55 and 107.38 deaths were averted for age group 40–49 years and 50–59 years respectively from screening mammography. The gain in life years from screening were 2466.14 years and 2566.93 years respectively for the age group of 40–49 years and 50–59 years.

**Table 2:** Results for life-years gained and cost–benefit analysis with the different costs for mammography

Category	Total cost (USD)	YLG	ICER	Cost/death averted	Number screened/death averted
The private institution only screening mammography					
Age 40–49 years	38,967,374	2466.14	15,800.95	496,078.17	1273.06
Age 50–59 years	38,585,063	2566.93	15,031.6	359,319	931.238
Government institution only screening mammography					
Age 40–49 years	8,656,833	2466.14	3510.275	110,206.7	1273.06
Age 50–59 years	8,278,038	2566.93	3224.87	77,088.2	931.238
Screening mammography with 50% of women screened at government and 50% at a private institution					
Age 40–49 years	24,014,173	2466.14	9737.55	305,714.92	1273.06
Age 50–59 years	23,633,597	2566.93	9206.93	220,085	931.238
Screening mammography with 90% of women screened at government and 10% at a private institution					
Age 40–49 years	11,768,715	2466.14	4739.343	148,793.85	1273.06
Age 50–59 years	11,308,740	2566.93	4405.54	105,311.26	931.238

*YLG* years of life gained, *ICER* incremental cost-effectiveness ratio, *USD* United States Dollars

All the policies evaluated the only policy for both age-groups the ICER value was above willingness to pay threshold with government institution screening mammography only policy yielding the lowest ICER value of 3510.275 and 3224.87 for age-group of 40–49 years and 50–59 years respectively. The lowest INMB loss was recorded in the government-only policy with − 1,730,678.81 USD and − 1,068,815.095 USD for age-group 40–49 years and 50–59 years respectively. 10%/90% private and government institutions screening mammography also yielded ICER of 4739.34 and 4405.54, both well above the cost-effectiveness threshold of 2808 USD for Ethiopia. The respective INMB values were − 4,842,560.81 USD and − 4,099,517.1 USD showing a net monetary loss.

Cost per life years gained were 110,206.7 USD/death averted and 77,088.2 USD/death averted for the age group of 40–49 years and 50–59 years respectively for government only policy. 1273.06 patients had to be screened to avert one death for the age group of 40–49 years of age with a lower number required, 931.238, for the age group of 50–59 years.

### Probabilistic sensitivity analysis

**Fig. 3:**
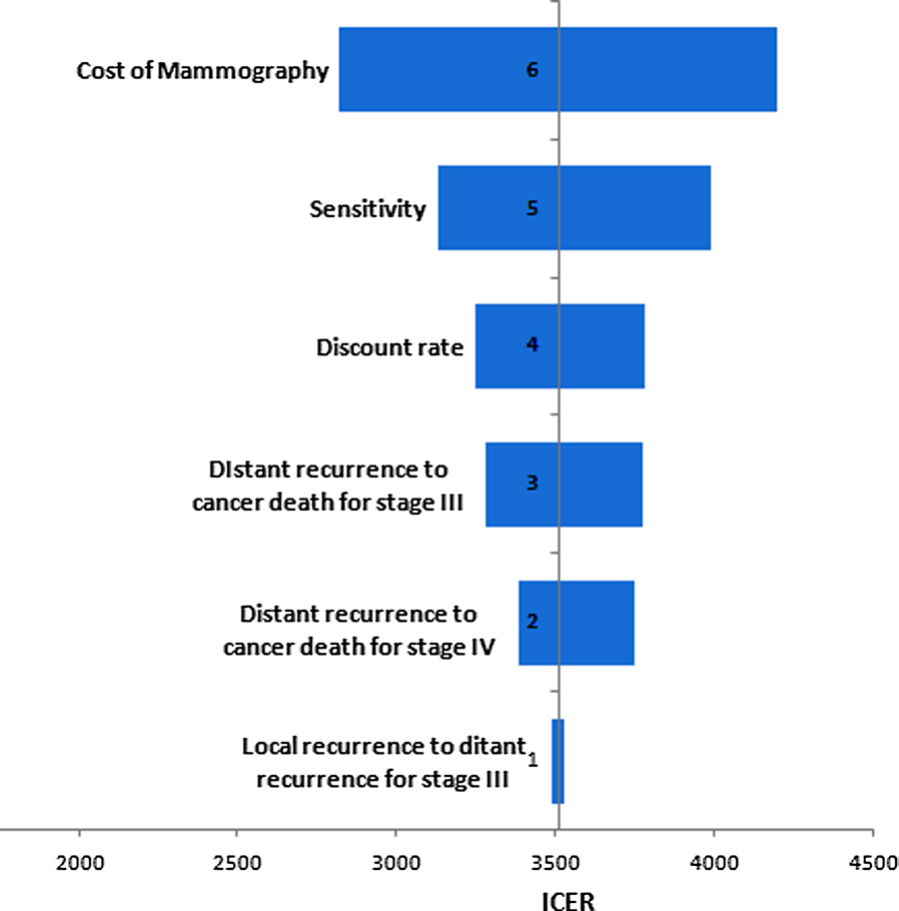
Impact of parameters on the ICER value for age 40–49 years

**Fig. 4:**
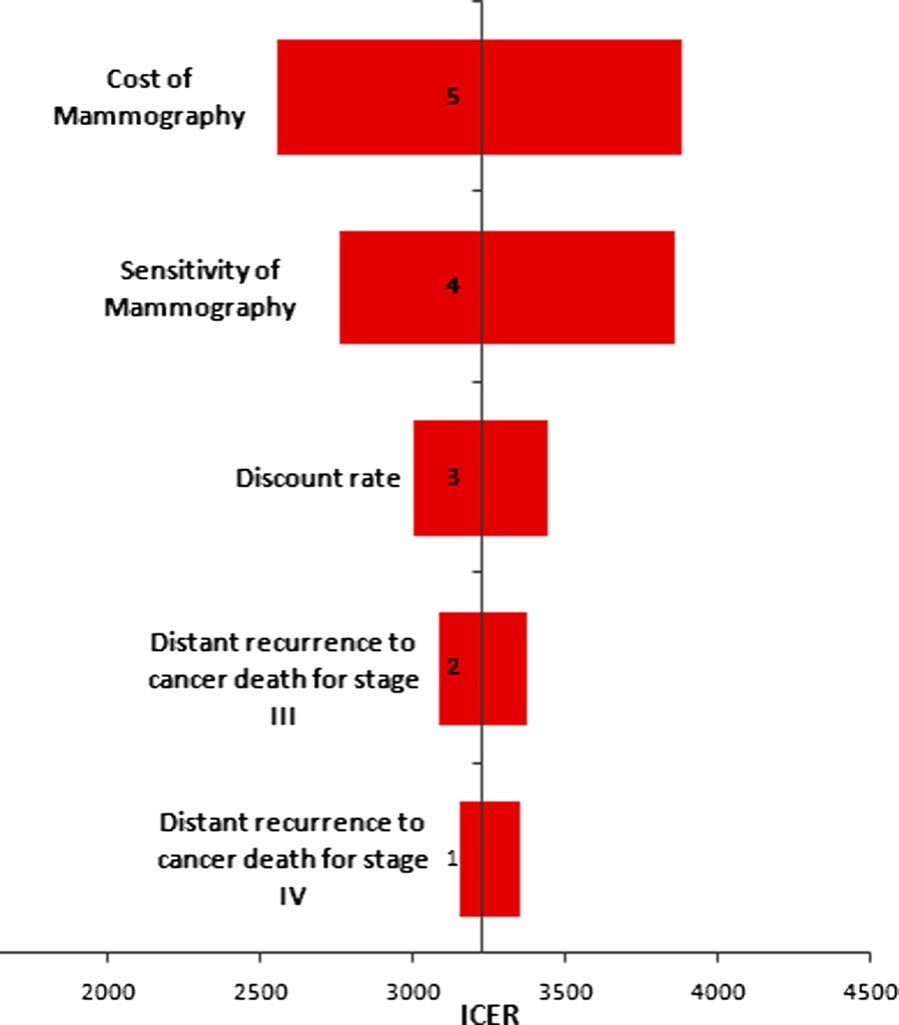
Impact of parameters on the ICER value for age 50–59 years

One-way sensitivity analysis was done using ICER as an output for both age groups with a government price policy and the values were presented in a tornado plot. (Figs. 3, 4) For both age categories, the price of screening mammography along with doctor visits and sensitivity of the screening mammography had the highest impact on output. The range of ICER for one-way sensitivity of the cost of mammography is 2822 to 4198.55 and for the sensitivity of the screening mammography was 3129.95 to 3991.5 for the age group of 40–49 years. The same effect is seen in the age group 50–59 years, with a range from one-way sensitivity analysis using the cost of mammography of 2563.7 to 3886.05, and for the sensitivity of the screening mammography, range of 2762.4 to 3866.9.

Different transition states for both age groups were evaluated and were found to affect ICER only in distant recurrence to death transition for stage III and IV in age-group of 50–59 years, and distant recurrence for stage III and IV, also local to distal recurrence transition for the age group between 40 and 49 years.

## Discussion

In this Markov simulation, annual mammography utilizing all four policies with an out-of-pocket payment plan was not shown to be cost-effective with INMB projecting net loss consistently. It required close to 110,206.7 USD and 77,088.2 USD to avert one death in each group of 40–49 years and 50–59 years respectively with the cheapest policy. Close to 1270 and just more than 930 women need to be screened to avert one death for age-group 40–49 years and 50–59 years respectively. The ICER was most affected by the price and the sensitivity of the screening mammography study. It is worth noting that 100% sensitivity for both age groups can theoretically yield acceptable cost per life years gained of 2527 and 2736 for age-group 40–49 years and 50–59 years respectively.

Except for Rwanda and Ghana, most African countries have no universal health care coverage despite the urge from WHO to progress away from service users out of pocket fees [25]. Ethiopia, like most African nations also, largely relies on out-of-pocket payment for health care services [26]. For this reason, screening mammography policies for the time being need to rely on an out-of-pocket payment plan.

Health care facilities in Ethiopia have significantly different price ranges based on the purpose of their establishment and the organization running the individual institutions. Price range increases progressively from Government Hospitals, Non-profit organizations, and finally private full profit health care institutions [27]. For this reason, separate simulations were required to analyze the possibility of private health sector involvement in the screening mammography policy and whether it is possible to achieve cost-effectiveness with either full, partial, or minimal involvement of the private sector in the screening program.

Costs per life years gained of this study with the use of 100% of private institutions with no government health facility involvement, 15,800.9 USD and 15,031.6 USD per life years gained were too high from a willingness to pay threshold. Understanding the underlying problem would be required to fully understand the output. First, similar studies have shown that these ICER values are not too high for annual screening mammography as the cost per life-years saved could range from 3400 USD to 83,830 USD based on institutions, regions, and countries of interest where the models were designed for [28]. Second, the price range of private health institutions, 42 USD, was not high as shown by a similar study from Canada, with a mean price of 150 USD and 137 USD in the US [15, 29]. Third, the GDP per capita of Ethiopia is very low and the cost-effectiveness ceiling has become too low to allow for a cost range of any amount as evidenced by negative INMB value even when the contribution of the private sector was reduced to only 10% in these models. So, the cost-effectiveness of screening mammography in any of the policies was not inherently ineffective, but rather relatively, and is likely to change with the change in the economy.

Government institutions only screening policy was the closest to favorable ICER with better value for age-group 50–59 years. This owed to the higher sensitivity of the test in the older age group compared to the younger cohort and is consistent with similar models done elsewhere [18]. In Sub-Saharan Africa, women may benefit more from younger age initiation of screening policy as, by the age of 50 years, most breast cancer cases have already been clinically diagnosed [7, 12, 13, 18]. In fact, one major drawback of any validated screening program is the higher younger patient proportion of breast cancer cases in low-income setups at the age younger than 40 years of age where validated screening protocol is lacking [7, 13, 18]. This would especially be invaluable for Ethiopia where more than 50% of breast cancer patients were found to be younger than 40 years of age [18]. In the younger population with a high risk of breast cancer, like BRCA mutation, the American Cancer Society recommends MRI along with mammography annually [30]. This obviously would be an unlikely alternative for average-risk group women owing to its high cost. Thus, for the age group below 40 years, a validated screening mechanism is needed on which a model can be generated for cost-effectiveness analysis.

This model intentionally omitted clinical breast examination, though it is a cheaper and more available alternative to screening mammography. The first reason for the omission was its notoriously low sensitivity at 54% [31]. Second, to date there is no evidence of its effect on reducing breast cancer-related mortality [32]. To this effect clinical breast examination is not recommended as a routine screening protocol by the American Cancer Society [30]. But with further study and validation for younger than 40 years of age women, annual clinical breast examination can be evaluated for its cost-effectiveness utilizing similar models.

## Conclusion

With the current cost of mammography in all policies evaluated using government and private institution prices of mammography, the life-years gained and the deaths averted could not justify the cost of screening mammography in Ethiopia. But with the development of the country, the GDP is expected to increase as well as the cost-effectiveness ceiling. For this reason, revisiting this model may be required and might reach a favorable conclusion. Furthermore, this model may achieve cost-effectiveness in some African countries with better GDP per capita and similar incidence of breast cancer provided that either health insurance is available or governmental subsidy is being implemented.

## Data Availability

Source data will be available upon reasonable request to the corresponding author.
